# The K–Cl Cotransporter KCC3 as an Independent Prognostic Factor in Human Esophageal Squamous Cell Carcinoma

**DOI:** 10.1155/2014/936401

**Published:** 2014-07-09

**Authors:** Atsushi Shiozaki, Kenichi Takemoto, Daisuke Ichikawa, Hitoshi Fujiwara, Hirotaka Konishi, Toshiyuki Kosuga, Shuhei Komatsu, Kazuma Okamoto, Mitsuo Kishimoto, Yoshinori Marunaka, Eigo Otsuji

**Affiliations:** ^1^Division of Digestive Surgery, Department of Surgery, Kyoto Prefectural University of Medicine, 465 Kajii-cho, Kamigyo-ku, Kyoto 602-8566, Japan; ^2^Department of Pathology, Kyoto Prefectural University of Medicine, Kyoto 602-8566, Japan; ^3^Departments of Molecular Cell Physiology and Bio-Ionomics, Graduate School of Medical Science, Kyoto Prefectural University of Medicine, Kyoto 602-8566, Japan; ^4^Japan Institute for Food Education and Health, St. Agnes' University, Kyoto 602-8013, Japan

## Abstract

The objectives of the present study were to investigate the role of K–Cl cotransporter 3 (KCC3) in the regulation of cellular invasion and the clinicopathological significance of its expression in esophageal squamous cell carcinoma (ESCC). Immunohistochemical analysis performed on 70 primary tumor samples obtained from ESCC patients showed that KCC3 was primarily found in the cytoplasm of carcinoma cells. Although the expression of KCC3 in the main tumor (MT) was related to several clinicopathological features, such as the pT and pN categories, it had no prognostic impact. KCC3 expression scores were compared between the MT and cancer nest (CN), and the survival rate of patients with a CN > MT score was lower than that of patients with a CN ≤ MT score. In addition, the survival rate of patients in whom KCC3 was expressed in the invasive front of tumor was lower than that of the patients without it. Furthermore, multivariate analysis demonstrated that the expression of KCC3 in the invasive front was one of the most important independent prognostic factors. The depletion of KCC3 using siRNAs inhibited cell migration and invasion in human ESCC cell lines. These results suggest that the expression of KCC3 in ESCC may affect cellular invasion and be related to a worse prognosis in patients with ESCC.

## 1. Introduction

The K–Cl cotransporter (KCC) mediates the coupled movement of K^+^ and Cl^−^ ions across the plasma membrane and is involved in the regulation of cell volume, transepithelial ion transport, and maintenance of intracellular Cl^−^ concentrations ([Cl^−^]_i_) [1, 2]. Four isoforms of the KCC have been identified and are termed KCC1, KCC2, KCC3, and KCC4 [3]. The four KCC isoforms share a common protein structure with 12 transmembrane regions in a central hydrophobic domain, together with hydrophilic N- and C-termini that may be cytoplasmic [4]. Although the expression of KCC1 is reportedly ubiquitous [5], that of KCC2 is restricted to neurons in the central nervous system [6]. KCC3 is expressed in the muscle, brain, lung, heart, and kidney [7], and KCC4 transcripts are the most abundant in the heart and kidney [4].

Several recent studies described the important roles of KCC in cancer development, tumor invasion, and possibly metastasis [8–11]. KCC3 was found to be important for cell-cycle progression, migration, and invasion in cervical carcinoma, ovarian cancer, breast cancer, and glioma [8, 9, 12, 13]. In addition, the overexpression of KCC3 downregulated the formation of the E-cadherin/*β*-catenin complex, and the subsequent upregulation of KCl cotransport activity was shown to benefit cancer cells in the epithelial-mesenchymal transition (EMT) [8]. However, the roles of KCC3 in the invasion of esophageal squamous cell carcinoma (ESCC) cells remain uncertain. Furthermore, the clinicopathological meaning of the expression of KCC3 in human ESCCs has not yet been evaluated.

The objectives of the present study were to investigate the roles of KCC3 in the cell migration and invasion of ESCC. Furthermore, we analyzed the expression of KCC3 in human ESCC samples and determined its relationships with the clinicopathological features and prognosis of ESCC patients. Our results revealed the important role of KCC3 in the tumor progression of ESCC.

## 2. Materials and Methods

### 2.1. Patients and Primary Tissue Samples

ESCC tumor samples were obtained from 70 patients with histologically proven primary ESCC who underwent esophagectomy (potentially curative R0 resection) at Kyoto Prefectural University of Medicine (Kyoto, Japan) between 1998 and 2007 and were analyzed retrospectively. These samples were embedded in paraffin 24 h after being fixed in formalin. Patient eligibility criteria included not having developed synchronous tumors or multiple metachronous tumors and not having received preoperative chemotherapy or radiation therapy. We excluded patients with noncuratively resected tumors or nonconsecutive data. All patients gave their written informed consent for inclusion in this study. Relevant clinicopathological and survival data were obtained from the hospital database. Staging was principally based on the seventh TNM staging system [14].

### 2.2. Immunohistochemistry

Paraffin sections (3 *μ*m thick) of tumor tissue were subjected to immunohistochemical staining for KCC3 using the avidin-biotin-peroxidase method. Briefly, paraffin sections were dewaxed in xylene and hydrated through a graded series of alcohols. Antigen retrieval was performed by heating the samples in Dako REAL Target Retrieval Solution (Glostrup, Denmark) for 40 min at 95°C. Endogenous peroxidase activity was quenched by incubating the sections for 30 min in 0.3% H_2_O_2_. Sections were incubated for one hour at room temperature with the following antibody: the KCC3 antibody (HPA034563; Atlas Antibodies AB, Stockholm, Sweden). The avidin-biotin-peroxidase complex system (Vectastain ABC Elite kit; Vector Laboratories, Burlingame, CA, USA) was used for color development with diaminobenzidine tetrahydrochloride. Sections were counterstained with hematoxylin. These sections were then dehydrated through a graded series of alcohols, cleared in xylene, and mounted. Control sections of known positive ESCC were included in each antibody run, and negative control sections were produced by omitting the primary antibody.

Immunohistochemical samples stained with KCC3 were graded semiquantitatively by considering both the staining intensity and percentage of positive tumor cells using an immunoreactive score (IRS) [15]. Staining intensity was scored as 0 (no staining), 1 (weak staining), 2 (moderate staining), or 3 (strong staining). The proportion of positive tumor cells was scored from 0 to 1.0. The score of each sample was calculated as the maximum multiplied product of the intensity and proportion scores (0 to 3.0).

### 2.3. Cell Culture

The human ESCC cell lines TE5 and TE9 were obtained from the Cell Resource Center for Biomedical Research at the Institute of Development, Aging, and Cancer (Tohoku University, Sendai, Japan) [16]. These cells were grown in RPMI-1640 medium (Nacalai Tesque, Kyoto, Japan) supplemented with 100 U/mL of penicillin, 100 *μ*g/mL of streptomycin, and 10% fetal bovine serum (FBS). Cells were cultured in flasks or dishes in a humidified incubator at 37°C under 5% CO_2_ in air.

### 2.4. Small Interfering RNA (siRNA) Transfection

Cells were transfected with 10 nmol/L KCC3 siRNA (Stealth RNAi siRNA #HSS HSS115159, HSS190762, HSS190763; Invitrogen, Carlsbad, CA) using the Lipofectamine RNAiMAX reagent (Invitrogen), according to the manufacturer's instructions. The medium containing siRNA was replaced with fresh medium after 24 h. We used three independent KCC3 siRNAs to exclude off target effects. The control siRNA provided (Stealth RNAi siRNA Negative Control; Invitrogen) was used as a negative control.

### 2.5. Real-Time Reverse Transcription-Polymerase Chain Reaction (RT-PCR)

Total RNA was extracted using an RNeasy kit (Qiagen, Valencia, CA). Messenger RNA (mRNA) expression was measured by quantitative real-time PCR (7300 Real-Time PCR System; Applied Biosystems, Foster City, CA) with TaqMan Gene Expression Assays (Applied Biosystems), according to the manufacturer's instructions. Expression levels were measured for the following gene: KCC3 (Hs00994548_m1) (Applied Biosystems). Expression was normalized for the KCC3 gene to the housekeeping gene beta-actin (ACTB, Hs01060665_g1; Applied Biosystems). Assays were performed in duplicate.

### 2.6. Analysis of Cell Migration and Invasion

The migration assay was conducted using a Cell Culture Insert with a pore size of 8 *μ*m (BD Biosciences, Bedford, MA). Biocoat Matrigel (BD Biosciences) was used to evaluate cell invasion potential. Briefly, cells (1.5 × 10^5^ cells per well) were seeded in the upper chamber in serum-free medium 24 h after siRNA transfection. The lower chamber contained medium with 10% FBS. The chambers were incubated for 48 h at 37°C in 5% CO_2_, and nonmigrated or noninvaded cells were then removed from the upper side of the membrane by scrubbing with cotton swabs. Migrated or invaded cells were fixed on the membrane and stained with Diff-Quick staining reagents (Sysmex, Kobe, Japan). The migrated or invaded cells on the lower side of the membrane were counted in four independent fields of view at 100x magnification of each insert. Each assay was performed in triplicate.

### 2.7. Statistical Analysis

Statistical analysis was carried out using Fisher's exact test to investigate correlations between clinicopathological parameters and KCC3 expression. Survival curves were constructed using the Kaplan-Meier method, and differences in survival were examined using the log-rank test. Multivariate analysis of the factors influencing survival was performed using the Cox proportional hazard model. Multiple comparisons were carried out using Dunnett's test after one-way ANOVA. Differences were considered significant when the associated *P* value was less than 0.05. All analyses were performed using statistical software (JMP, version 10; SAS Institute Inc., Cary, NC, USA). Correlation analyses were performed by creating Fit *Y* by *X* plots using JMP.

## 3. Results

### 3.1. KCC3 Protein Expression in Human ESCCs

**Figure 1:**
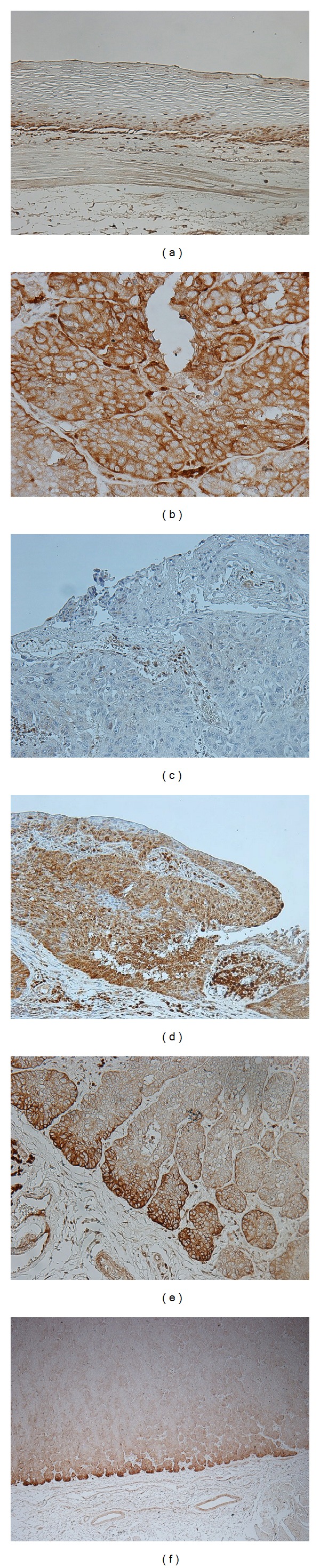
KCC3 protein expression in human esophageal squamous cell carcinoma (ESCC). (a) Immunohistochemical staining of noncancerous esophageal epithelia with the KCC3 antibody. Magnification: ×200. (b) Immunohistochemical staining of primary human ESCC samples with the KCC3 antibody. Magnification: ×400. (c) Immunohistochemical staining of primary human ESCC samples with the low grade expression of KCC3 in the main tumor (MT). Magnification: ×200. (d) Immunohistochemical staining of primary human ESCC samples with the high grade expression of KCC3 in the main tumor (MT). Magnification: ×200. (e) Immunohistochemical staining of primary human ESCC samples with the high grade expression of KCC3 in the cancer nest (CN). Magnification: ×200. (f) Immunohistochemical staining of primary human ESCC samples that expressed KCC3 in the invasive front of the tumor. Magnification: ×40.

An immunohistochemical investigation with the KCC3 antibody revealed the expression of KCC3 in the parabasal cell layer of normal esophageal mucosa (Figure 1(a)). We examined the expression of KCC3 in 70 primary tumor samples of human ESCC based on their immunohistochemical reactivity. The KCC3 protein was mostly expressed in the cytoplasm of carcinoma cells (Figure 1(b)). The KCC3 score in the main tumor (MT) varied widely between the tumors. The minimum KCC3 score was 0 while the maximum KCC3 score was 2.4 in MT (median = 0.725; mean ± standard error of the mean (SEM) = 0.780 ± 0.072). Regarding the expression of KCC3 in MT, we divided ESCC patients into 2 groups using the median staining score: a low grade KCC3 expression group with staining scores ≤0.725, *n* = 35, and a high grade KCC3 expression group with staining scores >0.725, *n* = 35. Figures 1(c) and 1(d) show the representative histopathological findings of low or high KCC3 expression samples. Correlations between the expression of KCC3 in MT and various clinicopathological parameters were analyzed (Table 1). We found correlations between the expression of KCC3 in MT and location of the primary tumor, the pT or pN category (Table 1).

**Figure 2:**
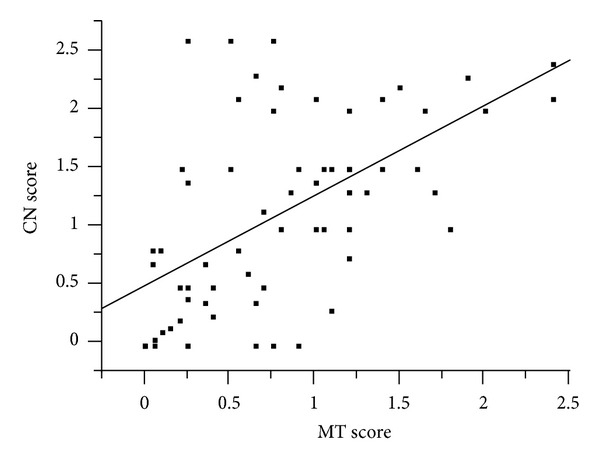
A correlation analysis of the relationship between the KCC3 score in main tumor (MT) and KCC3 score in the cancer nest (CN) was performed by producing Fit *Y* by *X* plots. The KCC3 score in CN was positively correlated with the KCC3 score in MT (*R* ^2^ = 0.3388, *P* < 0.0001).

We then focused on the expression of KCC3 in the cancer nest (CN) of ESCC (Figure 1(e)) and analyzed the KCC3 score in CN. The minimum KCC3 score was 0, and the maximum KCC3 score was 2.6 in CN (median = 1.000; mean ± SEM = 1.087 ± 0.096). The KCC3 score in CN was positively correlated with the KCC3 score in MT (*R* ^2^ = 0.3388, *P* < 0.0001) (Figure 2). Regarding the expression of KCC3 in CN, we divided ESCC patients into 2 groups using the median staining score, a low grade KCC3 expression group with staining scores ≤1.000, *n* = 36, and a high grade KCC3 expression group with staining scores >1.000, *n* = 34, and compared their clinicopathological features (Table 1). A correlation was found between the expression of KCC3 in CN and location of the primary tumor (Table 2). Regarding the comparison of KCC3 scores between MT and CN in each sample, we divided ESCC patients into 2 groups, CN > MT, *n* = 39, and CN ≤ MT, *n* = 31, and compared their clinicopathological features (Table 1). A correlation was not found between the comparison of KCC3 scores and clinicopathological features (Table 1).

Furthermore, we analyzed the localization of KCC3 expression in tumors. In 48 cases, the expression of KCC3 was found in the invasive front of the tumor (Figure 1(f)). Regarding the expression of KCC3 in the invasive front of the tumor, we divided ESCC patients into 2 groups, negative (*n* = 22) and positive (*n* = 48), and compared their clinicopathological features (Table 2). A correlation was found between the expression of KCC3 in the invasive front and the MT score, CN score, or their comparison (CN/MT) (Table 2). No correlation was found between the expression of KCC3 in the invasive front and any other clinicopathological parameter (Table 2).

### 3.2. Prognostic Impact of KCC3 Protein Expression for Patients with ESCC

**Figure 3:**
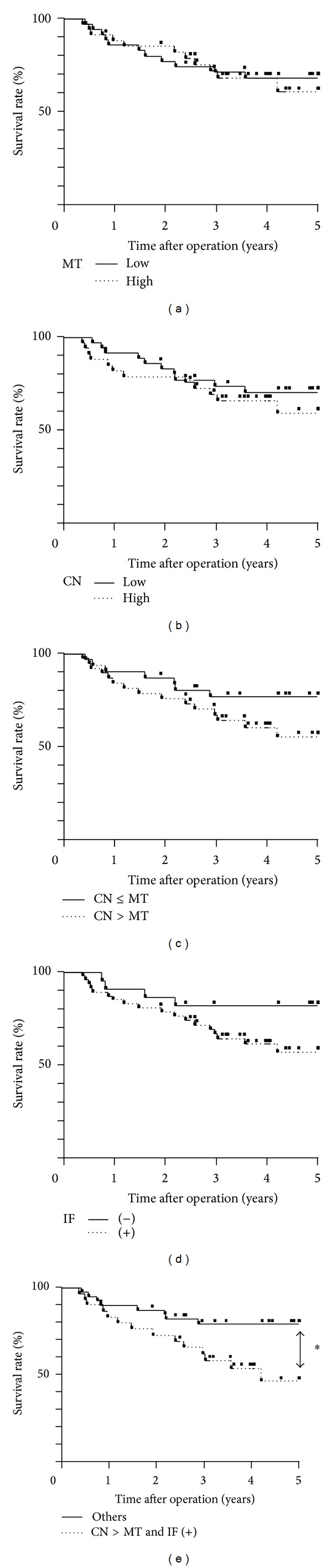
Survival curve of patients after curative resection for esophageal squamous cell carcinoma (ESCC) according to the expression of KCC3. (a) Patients were classified into two groups: low grade expression of KCC3 (*n* = 35) and high grade expression of KCC3 (*n* = 35) in the main tumor (MT). (b) Patients were classified into two groups: the low grade expression of KCC3 (*n* = 36) and high grade expression of KCC3 (*n* = 34) in the cancer nest (CN). (c) Patients were classified into two groups based on comparisons of the KCC3 score: CC ≤ MT (*n* = 31) and CN > MT (*n* = 39) in the cancer nest (CN). (d) Patients were classified into two groups based on the expression of KCC3 in the invasive front of the tumor: negative (*n* = 22) and positive (*n* = 48). (e) Patients were classified into two groups: patients with CN > MT and invasive front positive (*n* = 31) and others (*n* = 39). ^∗^ *P* < 0.05: log-rank test.

We determined the prognostic impact of the expression of KCC3 for patients with ESCC. Regarding the expression of KCC3 in MT, no significant difference was observed in the 5-year survival rate between patients with the high grade expression of KCC3 and those with the low grade expression of KCC3 in MT (Figure 3(a)). Similarly, no significant differences were observed in the 5-year survival rate between patients with the high grade expression of KCC3 and those with the low grade expression of KCC3 in CN (Figure 3(b)). Regarding comparisons of KCC3 score, the 5-year survival rate of the patients with CN > MT (55.5%) was lower than that of patients with CN ≤ MT (76.7%) (*P* = 0.133) (Figure 3(c)). The 5-year survival rate of the patients with KCC3 expression in the invasive front (57.1%) was lower than that of the patients without it (81.8%), although there was no statistical difference (*P* = 0.089) (Figure 3(d)). Interestingly, when patients were divided into 2 groups, CN > MT and invasive front positive, *n* = 31, and others, *n* = 39, the 5-year survival rate of patients with CN > MT and invasive front positive (46.1%) was significantly lower than that of other patients (79.0%) (*P* = 0.022) (Figure 3(e)).

Furthermore, we assessed which of the 13 variables studied (age, gender, location of the primary tumor, histological type, tumor size, lymphatic invasion, venous invasion, pT and pN category, KCC3 score in MT, KCC3 score in CN and CN/MT, and KCC3 expression in the invasive front) influenced survival following curative resection of esophageal cancer. Univariate analysis of survival after esophagectomy revealed that lymphatic invasion, venous invasion, and the pT and pN categories were found to be significant prognostic factors (*P* = 0.017, 0.017, 0.002, and 0.003, resp.) (Table 3). Multivariate analysis with variables whose *P* values were less than 0.100 in univariate analysis demonstrated that lymphatic invasion, pT and pN category, and KCC3 expression in invasive front were independent prognostic factors (*P* = 0.044, 0.015, 0.011, and 0.001, resp.) (Table 4). KCC3 expression in invasive front was the strongest prognostic factor of all clinicopathological features. These findings suggest that the expression of KCC3 might be a valuable prognostic factor for patients with ESCC.

### 3.3. KCC3 Controlled Cell Migration and Invasion in ESCC Cells

**Figure 4:**
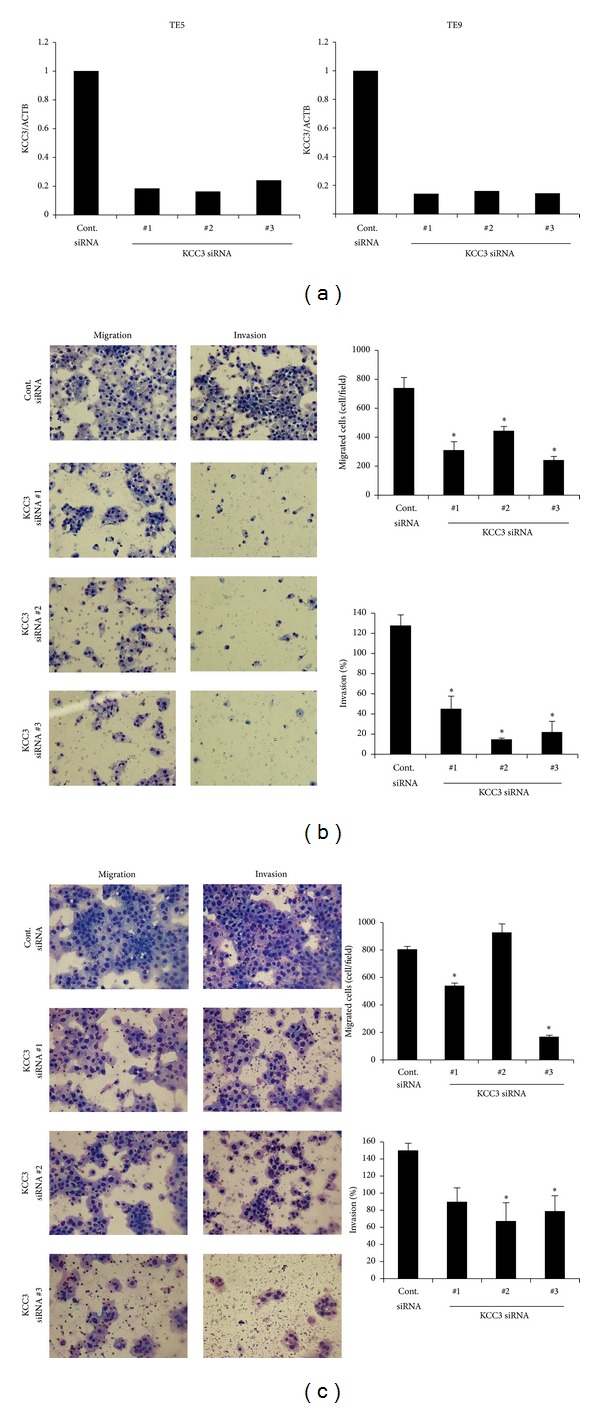
KCC3 controlled the cell migration and invasion of esophageal squamous cell carcinoma (ESCC) cells. (a) KCC3 siRNA effectively reduced KCC3 mRNA levels in both TE5 and TE9 cells. Three independent KCC3 siRNAs were investigated to exclude off target effects. (b) The downregulation of KCC3 significantly inhibited cell migration and invasion in TE5 cells. Cell migration and invasion were determined by the Boyden chamber assay. Mean ± SEM; *n* = 3. ^∗^ *P* < 0.05: Dunnett's test (ANOVA: migration; *P* < 0.0001, invasion; *P* < 0.0001). (c) The downregulation of KCC3 inhibited cell migration and invasion in TE9 cells. Cell migration and invasion were determined by the Boyden chamber assay. Mean ± SEM; *n* = 3. ^∗^ *P* < 0.05: Dunnett's test (ANOVA: migration; *P* < 0.0001, invasion; *P* = 0.0210).

We conducted knockdown experiments with KCC3 siRNA in ESCC cells and analyzed the effects of KCC3 knockdown on cell migration and invasion. We used three independent KCC3 siRNAs to exclude off target effects. All three KCC3 siRNAs effectively reduced KCC3 mRNA levels in both TE5 and TE9 cells (Figure 4(a)). In TE5 cells, all three KCC3 siRNAs significantly inhibited cell migration and invasion (Figure 4(b)). In TE9 cell, downregulation of KCC3 inhibited cell migration and invasion, too (Figure 4(c)). These results suggest that KCC3 plays an important role in regulating cell migration and invasion in ESCC cells.

## 4. Discussion

Recent studies have shown that ion channels and transporters play crucial roles in cellular functions, and their roles have been studied in cancer cells [17, 18]. Various types of ion channels, such as voltage-gated K^+^ channels, voltage-gated HERG channels, Ca_2_^+^ channels, and transient receptor potential channels, have been found to be expressed in gastrointestinal cancer cells and tissues and to regulate tumor behavior [19–22]. Regulators of intracellular pH such as anion exchanger, sodium-hydrogen exchanger, and carbonic anhydrases also related to tumor development of gastrointestinal cancer cells [23–25]. Furthermore, several reports have indicated that Cl^−^ channels/transporters, such as Cl^−^ channels, chloride intracellular channel (CLIC), KCC, and NKCC, play crucial roles in the tumorigenesis of colorectal, gastric, cervical, breast, lung, and prostate cancer cells [17, 18, 26]. We have also focused on and investigated transepithelial Cl^−^ transport in various types of cancer cells [11, 27–29].

In the present study, we investigated the KCC3 expression in ESCC and determined its relationships with clinicopathological features and prognosis. To the best of our knowledge, this is the first report examining KCC3 expression in human ESCC tissue. Our results showed that KCC3 expression in MT related to several clinicopathological features, such as the pT and pN categories. However, the expression of KCC3 in MT itself did not have a prognostic impact. Although these results may not be persuasive because of the limitation of a small sample size, they showed that KCC3 was expressed in MT of ESCC from an early stage. Regarding the expression of KCC in cancer tissue, previous studies demonstrated that KCC3 was abundant in cervical carcinoma and CN invaded deeply into stromal tissues whereas KCC4 was abundant in metastatic cervical and ovarian cancer tissues [8, 10]. Furthermore, both the progression-free and overall survival rates of patients with the high grade expression of KCC4 were significantly poorer than those of patients with the low grade expression of KCC4 in cervical cancer [10], which suggested a relationship between the expression pattern of KCC and clinical outcome. Therefore, we focused on the distribution of KCC3 in tumors and analyzed its expression in CN or the invasive front of the tumor. Although the expression of KCC3 in CN itself had no prognostic impact, the 5-year survival rate of patients with a CN > MT score was slightly lower than that of patients with a CN ≤ MT score. Furthermore, the 5-year survival rate of patients in whom KCC3 was expressed in the invasive front was lower than that of patients without it, and multivariate analysis revealed that the expression of KCC3 in the invasive front was the strongest prognostic factor of all clinicopathological features. These results suggest the role of KCC3 in cancer invasion as well as the importance of its distribution in tumors as a prognostic predictor. We have previously identified several prognostic biomarkers in human ESCC, such as Ki-67, antiphosphohistone H3, p21, and E2F5 [30–33]. The expressions of these cell-cycle related proteins were mainly analyzed in MT. On the other hand, we focused on the distribution of KCC3 in the present study and showed its prognostic impact via cellular invasion.

Recent studies have indicated the importance of KCC in the cell migration and invasion of glioma, cervical, ovarian, and breast cancer cells [8–10, 12, 13]. Regarding the mechanism by which KCC regulates tumor invasion, KCC3 was previously shown to downregulate the formation of the E-cadherin/*β*-catenin complex in order to promote EMT, which is important for cervical cancer cell invasiveness [8]. In addition, a previous study reported that the motor protein-dependent membrane trafficking of KCC4 was important for cancer cell invasion [10]. Our* in vitro* study also demonstrated the important roles of KCC3 in cell migration and invasion in ESCC cells. One possible mechanism by which KCC regulates the malignant behavior of cancer cells may be through the regulation of [Cl^−^]_i_ [11, 13]. Recent studies have shown that [Cl^−^]_i_ is a critical signal mediator for the regulation of various cellular functions [34–36]. For instance, we showed that [Cl^−^]_i_ could act as an important signal to control the gene expression of the epithelial Na^+^ channel via a tyrosine kinase in renal epithelial A6 cells [36]. We also previously reported that [Cl^−^]_i_ controlled cell-cycle progression in gastric and prostate cancer cells [27–29, 37, 38]. Shen et al. showed that an alteration in the [Cl^−^]_i_ concentration affected the activity of the retinoblastoma protein and cdc2 kinase, two key cell-cycle regulators that control progression from the G_1_ into the S phase and from the G_2_ into the M phase, respectively [13]. We considered KCC to be one of the important transporters that regulates [Cl^−^]_i_ in the steady state and previously showed that the blockage of KCC decreased [Cl^−^]_i_ in breast cancer cells [11]. Although this mechanism should be verified in more detail in further studies, these findings suggest that the changes induced in [Cl^−^]_i_ by KCC3 may be a critically important messenger that regulates cellular invasiveness in ESCC cells.

In summary, we found that KCC3 played a role in the cell migration and invasion of ESCC cells. An immunohistochemical analysis revealed that the expression of KCC3 in the invasive front of tumors was the strongest prognostic factor in patients with ESCC. A deeper understanding of the role of KCC3 may lead to its use as a crucial biomarker of tumor progression and/or a new therapeutic target for ESCC.

## Figures and Tables

**Table 1:** Relationships between the clinicopathological features of esophageal cancer and expression of KCC3 in the main tumor or cancer nest.

Variable	MT		*P* value	CN		*P* value	CN/MT		*P* value
	Low	High		Low	High		CN ≤ MT	CN > MT	
	(*n* = 35)	(*n* = 35)		(*n* = 36)	(*n* = 34)		(*n* = 31)	(*n* = 39)	
Age									
<60 years	11	11	1.000	11	11	1.000	6	16	0.071
≥60 years	24	24		25	23		25	23	
Gender									
Male	30	29	1.000	30	29	1.000	25	34	0.520
Female	5	6		6	5		6	5	
Location of the primary tumor									
Ut-Mt	18	29	0.0101^∗^	19	28	0.0112^∗^	21	26	1.000
Lt-Ae	17	6		17	6		10	13	
Histological type									
Well/moderately differentiated SCC	22	27	0.297	26	23	0.796	22	27	1.000
Poorly differentiated SCC	13	8		10	11		9	12	
Tumor size									
<50 mm	22	27	0.2968	24	25	0.6069	24	25	0.2967
≥50 mm	13	8		12	9		7	14	
Lymphatic invasion									
Negative	15	18	0.6324	20	13	0.1606	18	15	0.1482
Positive	20	17		16	21		13	24	
Venous invasion									
Negative	19	21	0.8094	20	20	0.8133	18	22	1.000
Positive	16	14		16	14		13	17	
pT									
pT1	10	23	0.0037^∗^	14	19	0.2309	18	15	0.1482
pT2-3	25	12		22	15		13	24	
pN									
pN0	11	22	0.0160^∗^	15	18	0.473	16	17	0.631
pN1-3	24	13		21	16		15	22	

MT: main tumor; CN: cancer nest; Ut: upper thoracic esophagus; Mt: middle thoracic esophagus; Lt: lower thoracic esophagus; Ae: abdominal esophagus; SCC: squamous cell carcinoma; pT: pathological T stage; pN: pathological N stage.

^∗^ *P* < 0.05: Fisher's exact test.

**Table 2:** Relationships between the clinicopathological features of esophageal cancer and expression of KCC3 in the invasive front of the tumor.

Variable	Invasive front		*P* value
	Negative	Positive	
	(*n* = 22)	(*n* = 48)	
Age			
<60 years	6	16	0.783
≥60 years	16	32	
Gender			
Male	21	38	0.154
Female	1	10	
Location of the primary tumor			
Ut-Mt	12	35	0.172
Lt-Ae	10	13	
Histological type			
Well/moderately differentiated SCC	15	34	1.000
Poorly differentiated SCC	7	14	
Tumor size			
<50 mm	14	35	0.575
≥50 mm	8	13	
Lymphatic invasion			
Negative	10	23	1.000
Positive	12	25	
Venous invasion			
Negative	13	27	1.000
Positive	9	21	
pT			
pT1	8	25	0.3035
pT2-3	14	23	
pN			
pN0	8	25	0.3035
pN1-3	14	23	
MT			
Low	20	15	<0.0001∗
High	2	33	
CN			
Low	19	17	<0.0001∗
High	3	31	
CN/MT			
CN ≤ MT	14	17	0.0385∗
CN > MT	8	31	

Ut: upper thoracic esophagus; Mt: middle thoracic esophagus; Lt: lower thoracic esophagus; Ae: abdominal esophagus; SCC: squamous cell carcinoma; pT: pathological T stage; pN: pathological N stage; MT: main tumor; CN: cancer nest.

^∗^ *P* < 0.05: Fisher's exact test.

**Table 3:** Five-year survival rate of patients with esophageal cancer according to various clinicopathological parameters.

Variables	5-year survival rate (%)	*P* value
Age		
<60 years	53.13	0.1179
≥60 years	72.2	
Gender		
Male	65.35	0.8597
Female	68.57	
Location of the primary tumor		
Ut-Mt	73.63	0.0564
Lt-Ae	50.82	
Histological type		
Well/moderately differentiated SCC	71.94	0.2301
Poorly differentiated SCC	53.43	
Tumor size		
<50 mm	68.68	0.2809
≥50 mm	59.52	
Lymphatic invasion		
Negative	78.4	0.0168^∗^
Positive	54.78	
Venous invasion		
Negative	78.18	0.0169^∗^
Positive	48.7	
pT		
pT1	82.22	0.0024^∗^
pT2-3	50.68	
pN		
pN0	82.72	0.0029^∗^
pN1-3	49.68	
MT		
Low	68.21	0.7838
High	60.56	
CN		
Low	70.48	0.4151
High	59.25	
CN/MT		
CN ≤ MT	76.74	0.1329
CN > MT	55.48	
Invasive front		
Negative	81.82	0.0887
Positive	57.07	

Ut: upper thoracic esophagus; Mt: middle thoracic esophagus; Lt: lower thoracic esophagus; Ae: abdominal esophagus; SCC: squamous cell carcinoma; pT: pathological T stage; pN: pathological N stage; MT: main tumor; CN: cancer nest.

^∗^ *P* < 0.05: log-rank test.

**Table 4:** Prognostic factors of esophageal cancer according to multivariate analysis.

Variables	Risk ratio	95% CI	*P* value
Location of the primary tumor			
Ut-Mt	Ref.	0.861452–2.20629	0.1813
Lt-Ae	1.37267		
Lymphatic invasion			
Negative	Ref.	1.012886–2.725895	0.0437^∗^
Positive	1.605417		
Venous invasion			
Negative	Ref.	0.954510–2.395284	0.08
Positive	1.483897		
pT			
pT1	Ref.	1.122531–3.250204	0.0146^∗^
pT2-3	1.834223		
pN			
pN0	Ref.	1.152392–3.450263	0.0110^∗^
pN1-3	1.911249		
Invasive front			
Negative	Ref.	1.357757–4.524146	0.0014^∗^
Positive	2.332559		

Ut: upper thoracic esophagus; Mt: middle thoracic esophagus; Lt: lower thoracic esophagus; Ae: abdominal esophagus; pT: pathological T stage; pN: pathological N stage; Ref.: referent.

^∗^ *P* < 0.05: Cox's proportional hazards model; 95% CI: 95% confidence interval.
